# Transcriptome Analysis of Drought Resistance in Japanese Lawn Grass (*Zoysia japonica* Steud.)

**DOI:** 10.3390/cimb48020209

**Published:** 2026-02-14

**Authors:** Ruijia Zhao, Lei Xu, Xinzi Wang, Yixuan Wei, Jian Chen, Yu Chen, Jun Liu

**Affiliations:** College of Agro-Grassland Science, Nanjing Agricultural University, Nanjing 210095, China; zrj200418@163.com (R.Z.); xul58700901@163.com (L.X.);

**Keywords:** turfgrass, drought stress, MAPK

## Abstract

With the intensification of global climate change, the increasing frequency and severity of extreme weather events seriously affected agroecosystems and human health. *Zoysia japonica* Steud. (*Z. japonica*) is a warm season turfgrass with outstanding drought tolerance; therefore, gaining insight into the breeding and ecological restoration of drought-tolerant lawn grass species is of great significance. This study aimed to investigate the adaptive strategies of drought-resistant *z047* and *z388* by integrating transcriptome analysis and experimental physiological measurements in a drought field. Physiological experiments have demonstrated that *z047* plants exhibited a stronger water retention capacity, lower cell membrane damage, and higher above-ground biomass. In addition, the relative water content and permanent wilting coefficient of *z047* plants were superior to wild type plants. Our results verified that there were 108 and 208 significantly differentially expressed genes (DEGs) (fold change (FC) ≥ 4, *p* < 0.01) screened from z047 plants under drought stress for 7 and 14 days, respectively. Moreover, remarkable upregulation of *MAPKKK17* and *MAPKKK16* genes involved in the MAPK signalling pathway may be closely related to their drought tolerance. Collectively, this study reveals the molecular and physiological synergistic mechanism of drought tolerance in *Z. japonica*, thus providing a theoretical basis for molecular breeding of drought-tolerant plant cultivars and ecological restoration in arid areas.

## 1. Introduction

The increasing frequency and severity of extreme weather events seriously affected agroecosystems. Among which, drought is one of the most important abiotic stress factors that cause adverse effects on plant cell gene expression, morphology, physiology, and function, posing a recurrent and severe meteorological challenge to significant shifts in plant species distribution and reductions in agricultural yields [1,2,3] Recently, accumulating evidence has confirmed that the deficit and limitation of available water adversely affect plant growth; however, the degree of impact varies among plant species depending on their phenological, morphological, physiological, and biochemical characteristics, as well as the methods of cultivation [4,5,6]. Furthermore, drought stress degrades plant metabolism and physiological processes, resulting in decreased growth and yield losses, ranging from 30% to 90% according to the crop species and stage of maturity [7]. The strong root system is the very organ where plants first sense and transfer signal to the above-ground plant organs [8,9]. Subsequently, plants undergo a process of adaptation involving the morphological characteristics (such as decreased stomatal area and metaxylem vessel size, and increased length of root) and biochemical parameter changes (such as a total reduction in chlorophyll content and higher production of reactive oxygen species) via genetic variation [10,11,12], leading to the production of corresponding proteins [13,14]. Based on a proper understanding of the physiological, biochemical, and molecular basis of drought tolerance, it is possible to develop drought-tolerant and high-yielding varieties suitable for water-limiting environments. Therefore, illustrating the molecular mechanisms underlying the plant structural and functional genomics may strongly provide a sustainable productivity in agricultural production [15]. As a result, plants can perceive stimuli from their environment and activate defence pathways via various modulating networks to cope with drought stress [16], consequently facilitating the breeding of drought-resistant varieties [17,18,19].

Zoysia is a kind of warm-season grass and there are three principal species of zoysia grass used for turf, namely *Zoysia japonica* (*Zoysia japonica* Steud.), *Zoysia matrella*, and *Zoysia tenuifolia,* with slightly different appearances and characteristics. Among these three most important commercial species, *Z.japonica* exhibits many excellent characteristics, including strong resistance to abiotic stress, disease, and insects, partly due to their well-developed rhizomes and stolons [20,21,22,23]. Despite numerous studies having documented the genetic and physiological response to drought stress in *Z. japonica* [20,24], there has been little focus on the development and improvement of drought-tolerant cultivars using molecular and biochemical approaches. Herein, this study aimed to investigate the adaptive strategies on improving the drought resistance by integrating transcriptome analysis and experimental physiological measurements in *Z. japonica*.

## 2. Material and Methods

### 2.1. Experimental Materials and Processing

Mutants of Japanese Zoysia grass (*Zoysia japonica* Steud.) were obtained by using ^60^Co-γ radiation in the early stage, and planted in fields. After observation, 5 strong drought-tolerant plants were obtained by examining 24 agronomic traits. On the basis of this, the drought-tolerant *z047* and *z388* were selected, and those without radiation mutagenesis were used as a wild type (WT) control. All plants were grown in a diameter of 110 mm and a length of 30 cm polyvinyl chloride (PVC) pipe which was filled with a mixture of peat, vermiculite, and organic fertilizer at a ratio of 3:1:1:1 in a glass greenhouse with a 16 h photoperiod at a temperature of 20~25 °C and 60% relative humidity at Nanjing Agricultural University. After germination, they were each transplanted to 1 of 11 separate pots and transferred to the experiment field, where they were grown, watered to field capacity (≥75%) every 2 days and fertilized once a week for one month under natural conditions. Thereafter, plants were grown under drought condition for 25 days and then rehydration recovery for 7 days, with a total of 33 plant samples.

### 2.2. Analysis of Leaf Relative Water Contents

According to a previous study [25], the *Z. japonica* leaves were taken to measure relative water contents. Briefly, three kinds of fresh *Z. japonica* leaves for each sample were collected to measure their weight. Afterward, the leaves were separately immersed in a stoppered test tube containing distilled water for 12 h and then the weight of fully turgid leaves were measured. Thereafter, the leaves were marked, dried in an oven (70 ± 2 °C, 24 h), and then their dry weight was calculated on a balance after cooling down to room temperature and completely volatilizing all moisture. The relative water content (%) = [(Fresh weight − Dry weight)/(Fully turgid weight − Dry weight)] × 100.

### 2.3. Analysis of Leaf Electrolyte Leakage (EL)

*Z. japonica* leaves were cut and cleaned with distilled water. After removing excessive water from the surface of cleaned leaves with paper towels, four discs approximately 5 mm in diameter were excised from each sample, weighed, and subsequently soaked in glass tubes with 20 mL distilled water, which were placed in an incubator for 24 h at 20 °C in the dark. Electrical conductivity (EC) at time point 1, namely EC1, was determined on a WTW 3.15i conductivity meter at 25 °C. Subsequently, all tubes were soaked in a thermostatically controlled water bath at 90 °C for 40 min and afterwards the colour measurements were again determined for EC2 at 25 °C after cooling down in an incubator. At last, the EL was calculated using EC values as follows: EL = (EC1/EC2) × 100.

### 2.4. Analysis of Leaf Chlorophyll Content

Fresh leaves approximately 0.05 g were taken from each sample, cut into small sections of nearly 5 mm, and then placed them into centrifuge tubes filled with 10 mL ethanol (95%), respectively. After sealing the mouth of the bottle, the test tube was wrapped in 2 layers of black plastic bag, tied with a rubber band, and stored in a dark place for 48 h. The mixture was filtered in a 25 mL volumetric flask and 0.8~1.0 mL filtrate was added to the cuvette to measure the absorbance at the wavelengths of 663 nm and 645 nm, with 95% ethanol as the blank. Chlorophyll a content (mg/L) = 12.21 × A _663 nm_ − 2.81 × A_645 nm_; Chlorophyll b content (mg/L) =20.13 × A_645 nm_ − 5.03 × A_663 nm_.

### 2.5. Library Construction and Sequencing

To sequence the genes related to drought tolerance, the drought-tolerant *z047* and wild type plants were placed under different stress conditions and then sent to Baimaike Biotechnology Co., Ltd. (Beijing, China) for RNA extraction and library construction. The libraries were subjected to double-ended sequencing.

### 2.6. Screening and Identification of Differentially Expressed Genes (DEGs)

Transcriptome data were separately sampled from *z047* plants after 7-day and 14-day induction of drought, and then compared with WT plants, respectively. TBTools software (version 2.376) was used for analysis, and the DEGs were identified based on the following thresholds: Q-value (adjusted *p*-value) ≤ 0.01 and fold change (FC) ≥ 4 [26]. Simultaneously, an in-depth comparative transcriptome analysis of *Z. japonica* genes under drought stress, along with enrichment analysis and pathway analysis, was performed using Gene Ontology (GO) and Kyoto Encyclopedia of Genes and Genomes (KEGG). The item was sorted in descending order with *p* < 0.01, and the top 20 enrichment analysis items were selected for subsequent analysis.

### 2.7. Statistical Analysis

Data were expressed as the mean and standard deviation, and all experiments were performed at least in triplicate. Comparison between two groups was performed using the student’s *t*-test. Differences among groups were analyzed by a one-way analysis of variance (ANOVA) and Duncan’s test using SPSS statistics 26.0 (SPSS Inc., Chicago, IL, USA). A *p* value less than 0.05 was considered statistically significant.

## 3. Results and Discussion

### 3.1. Morphological Responses of Z. japonica Under Different Stress Conditions

**Figure 1:**
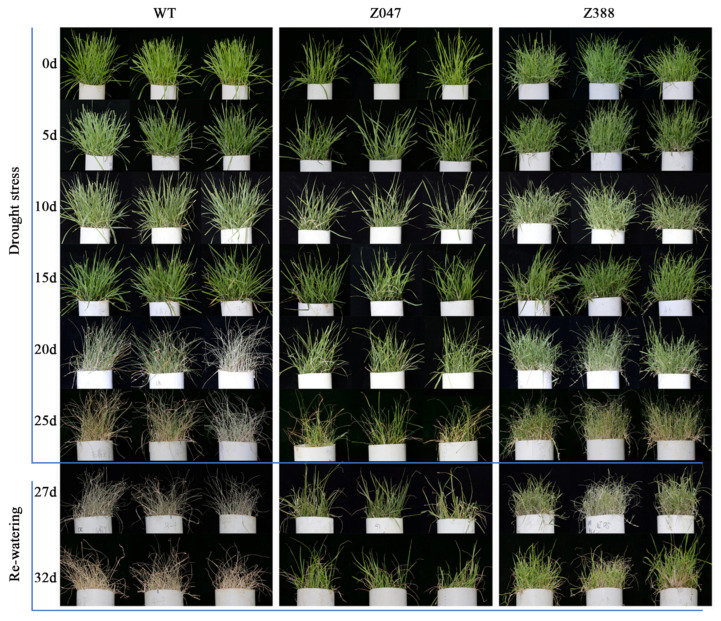
Morphologies of three *Z. japonica* species under drought stress. All *Z. japonica* were under drought stress from 0 to 25 days and then rehydration recovery from 27 to 32 days.

Drought is one of the deleterious abiotic stress factors that constrain crop growth and development [27]. The direct impact of drought was first observed on the morphological and physiological changes in plant leaves; these are the primary source of photosynthetic productivity. Under drought stress, all grasses initially grew well and had green blades in *z047* and *z388* and WT, but there were no significant differences among them. During 0–15 DAD (days after drought), all grasses grew slowly and turned a lighter shade of green or blue-grey, implying the first visible signs of water deficiency. At 20–25 DAD, most grasses progressed to a more severe yellow with a lighter shade of green or blue-grey in *z047* and *z388*, especially in *z388*, whereas all grasses withered in WT. Simultaneously, some blades appeared limp, curled inward, or folded in half lengthwise in all groups. Thereafter, a deep, thorough watering rehydrated the grass roots and gradually revived the grasses in *z047* and *z388* but not in WT from day 25 to day 27. Of note, rehydration recovery gradually revived parts of withered grasses in *z047* and *z388*, especially in *z047*, but it did not revive the grass in WT (Figure 1). These observations are similar to those reported in previous studies in other plant species under drought and after rehydration recovery [28,29]. Accordingly, these results indicate substantial morphological and physiological responses of *Z. japonica* under drought and after rehydration recovery, and the *z047* plants may be useful for exploring the molecular breeding of drought-tolerant grass cultivars and ecological restoration in arid areas.

### 3.2. Soil Water Content in the Pots of Z. japonica Under Different Stress Conditions

**Figure 2:**
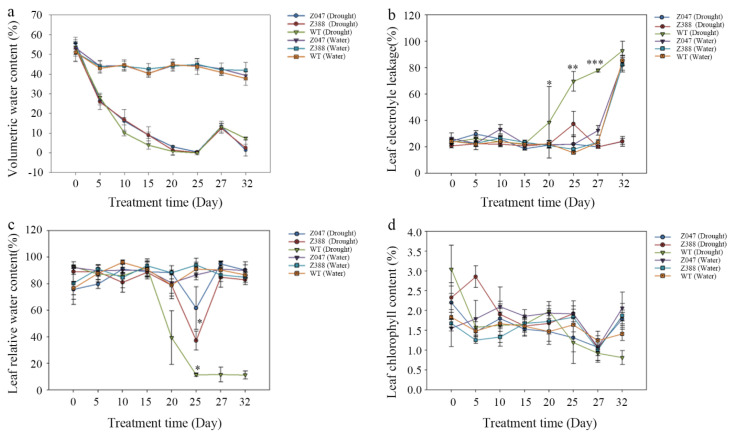
Physiological changes in *Z. japonica* under different stress conditions during a 32-day sampling period. (**a**) Volumetric water content in soil of *Z. japonica*. (**b**) Electrolyte leakage of *Z. japonica*. (**c**) Relative water content of *Z. japonica leaves*. (**d**) Chlorophyll content of *Z. japonica* leaves. *z047* (Drought), *z047* plants under drought stress; *z388* (Drought), *z388* plant under drought stress; WT (Drought), wild type plant under drought stress; *z047* (Water), *z047* plants under well-watered condition; *z388* (Water), *z388* plants under well-watered conditions; WT (Water), wild type plants under well-watered conditions. * *p* < 0.05, ** *p* < 0.01, *** *p* < 0.001.

Water content in soil affects growth, development, and physiological processes of plants [30], and it is one of the critical indicators in agricultural systems. Measuring the volumetric water content (VWC) in soils is the most frequent application of time domain reflectometry in soil science and soil hydrology [31]. As reported, 50–60 wt% soil water content (SWC) was suitable for seedling cultivation; however, 10–20 wt% SWC, 30–40 wt% SWC, and 70–80 wt% SWC had negative effects on seedling growth, and seedlings adapt to unfavourable water condition by morphological and physiological responses under 10–20 wt% SWC or 70–80 wt% SWC [32]. In this study, the initial VWC in soil showed a downward trend in all groups from day 0 to day 25, and it ranged from 54.0% to 0.33% in the pots of *Z. japonica* under drought stress for 25 days (Figure 2a). After rehydration recovery, the VWC presented an upward trend from day 25 and reached the highest point on day 27, ranging from 12.5% to 13.38%. Afterward, the VWC decreased slightly again, ranging from 1.35% to 7.35% on day 32 (Figure 2a), but there were no significant differences among them, implying the same level of drought stress for all treatments in our current study. Of note, extreme experimental drought resulted in a significant decrease in VWC and the negative impacts did not persist in the grasslands, as evidenced by the fact that VWC was similar to ambient conditions after the removal of drought [33]. Within our study system, the VWC showed a downward trend from day 0 to day 5 in all groups, and subsequently, it remained in a dynamic equilibrium, ranging from 37.75% to 44.93% from day 5 to day 32 under well-watered conditions (Figure 2a). Accordingly, the initial VWC is nearly 54% in this study area.

### 3.3. Electrolyte Leakage Rate (ELR) of Z. japonica Under Different Stress Conditions

Leaf cellular membrane stability determined by electrolyte leakage was related to more leaf turgor loss point (*pi*(tlp)), which is a chief parameter for characterizing relative drought tolerance among species and signifies the point at which leaf cells lose their turgor, or wilt [34,35]. The plant species with greater drought tolerance also showed greater membrane stability, suggesting membrane integrity is a potential mechanism associated with maintenance of leaf drought tolerance [34]. Our results indicated that the ELR of *Z. japonica* showed tiny fluctuations from day 0 to day 15, but there was no significant difference among them (*p* > 0.05). Interestingly, under persistent drought stress, there was no significant difference in the ELR in *z047* and *z388* compared with WT (18.2% vs. 22.40%; 20.81% vs. 22.40%, all *p* > 0.05) on day 15. From day 15 to day 20, the ELR increased slightly and reached 38.61% in WT, with a relative steady state in *z047* and *z388*. Afterward, the ELR increased remarkably in WT group, with a mild rise in *z388* and a steady state in *z047* from day 20 to day 25. Notably, the ELR was lower in *z388* than that in WT (37.29% vs. 69.65%, *p* < 0.05). Under well-watered conditions, the ELR did not obviously fluctuate and remained relatively stable in all groups, ranging from 15.51% to 33.2% (Figure 2b). All these data imply that *z047* showed many tolerant abilities to drought stress.

### 3.4. Relative Water Content of Z. japonica Leaves Under Different Stress Conditions

In plants, stress can be monitored by changes in water status, plant growth, and electrolyte leakage [36]. In this study, RWC held steady in all groups, with a relatively small fluctuation from day 0 to day 15. Under drought stress, there was no significant difference in RWC in *z047* and *z388* compared with WT group (88.93% vs. 92.06%; 88.54% vs. 92.06%, all *p* > 0.05) on day 15. From day 15 to day 20, the RWC decreased dramatically and reached 39.36% on day 20 in WT group, with a minimal fluctuation in *z047* and *z388*. Thereafter, the RWC declined sharply and dropped to 11.44% in WT group, which was considerably lower than that in *z388* (38.64%) and *z047* (61.62%) on day 25 (all *p* < 0.05). Interestingly, the RWC stayed constantly at a low level in WT after rehydration recovery, whereas it increased rapidly in *z047* and *z388* from day 25 to day 32, fluctuating from 82.96% to 90.26%, and subsequently tended to stay constant. On the contrary, the RWC of WT leaves showed a high level and remained relatively stable under well-watered conditions, ranging from 96.50% to 96.13% (Figure 2c). Collectively, the mutant *z047* and *z388* plants have a high RWC during persistent drought stress and they also have higher RWC after rehydration recovery, implying a resistant ability against drought stress.

### 3.5. Leaf Chlorophyll Content of Z. japonica Under Different Stress Conditions

Chlorophyll mainly composed of chlorophyll a (Chl a) and chlorophyll b (Chl b) plays a pivotal role in photosynthesis [37]. Chlorophyll content is one of valuable diagnostic indicators for early identification and assessment of overall health of vegetation [38]. Also, many studies have shown that chlorophyll content is an important factor that should be examined to determine plant stress conditions [21,39]. Under persistent drought stress, there was a sharp downward trend of leaf chlorophyll content in *z047* and WT, but there was a slow rising of chlorophyll content in *z388* from day 0 to day 5. On day 5, the chlorophyll content dropped to 1.58 mg/g in WT, 1.47 mg/g in *z047*, and 2.85 mg/g in *z388* (Figure 2d). From day 5 to day 10, the chlorophyll content remained stable in WT, increased slowly in *z047*, and decreased sharply in *z388*. From day 10 to day 20, the chlorophyll content increased slowly in WT, decreased slowly in *z047*, and showed a trend of decreasing first and then rising in *z388*. From day 20 to day 32, the chlorophyll content significantly decreased in WT, presented a trend of decrease first and then rose in *z047*, and slowly elevated in *z388* from day 20 to day 25, followed by a trend of decreasing and then rising. On day 32, the chlorophyll content was 0.81 mg/g in WT, 2.06 mg/g in *z047*, and 1.85 mg/g in *z388*. In contrast, the chlorophyll content slightly fluctuated in all groups, ranging from 1.01 mg/g to 2.10 mg/g under well-watered conditions (Figure 2d). As a result, the leaf chlorophyll content of *Z. japonica* shows dynamic changes under persistent drought stress for 25 days but the *z047* owns a high level of chlorophyll content after rehydration recovery, implying that *z047* has a strong ability of drought-tolerance.

### 3.6. Permanent Wilting Coefficient and Above-Ground Biomass of Z. japonica Under Different Stress Conditions

**Figure 3:**
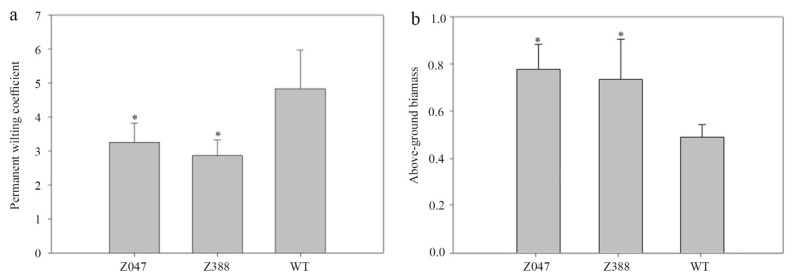
Permanent wilting coefficient (**a**) and above-ground biomass (**b**) of *Z. japonica* under drought stress. * *p* < 0.05.

Water content between the field capacity and permanent wilting coefficient (PWC) is considered to be available to plants for uptake, while the field capacity minus the permanent wilting coefficient conveys the maximum available water content [40,41]. In this study, the PWC under drought stress was lower in *z047* and *z388* groups than that in WT group (*p* < 0.05), but there was no significant difference between *z047* and *z388* (*p* > 0.05). Numerous studies have shown that under a limited water supply, a larger root biomass is associated with an increased above-ground biomass [42]. However, it has been demonstrated that understanding of plant responses to biotic and abiotic drivers is largely based on above-ground plant traits [43]; our relative above-ground biomass under drought stress was significantly higher in *z047* and *z388* than that in WT group (Figure 3). Collectively, all these data indicate that the tolerance of *z047* and *z388* plants to drought stress is superior to their non-mutated parent plants.

### 3.7. Analysis of DEGs and Gene Ontology (GO) Functional Enrichment of Z. japonica Under Drought Stress

A total of 27,372 reads were obtained from *z047* plants under drought stress for 7 and 14 days, and 108 remarkable DEGs comprising 77 up-regulated and 31 down-regulated genes were screened. Under drought stress for 14 days, a total of 30,967 were obtained and, subsequently, 208 DEGs, including 73 up-regulated and 135 down-regulated genes, were identified.

**Figure 4:**
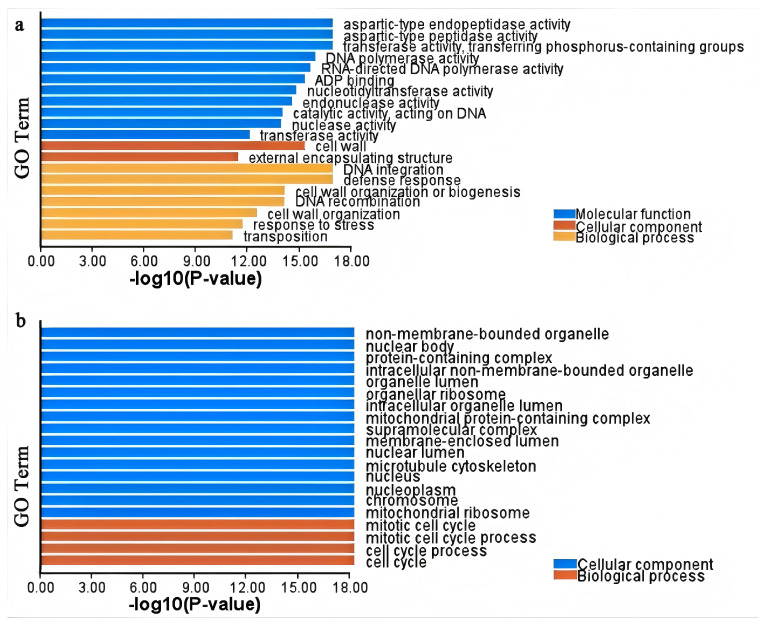
Analysis of Gene Ontology functional enrichment. (**a**) z047 plants under drought stress for 7 days; (**b**) *z047* plants under drought stress for 14 days.

Next, GO function enrichment analysis of DGEs histogram showed that under drought stress for 7 days, the number distribution of DGEs was mainly enriched in 11 molecular functions (e.g., aspartic-type endopeptidase activity, aspartic-type peptidase activity, and transferase activity containing transferring phosphorus-containing groups), 2 cellular components, and 7 biological processes in *z047* (Figure 4). Furthermore, the number distribution of DEGs was mainly enriched in 16 cellular components, and 4 biological processes in *z047* under drought stress for 14 days. All these data imply strong molecular functions and biological processes in the early stage and weak biological processes in the late stages of *z047*.

### 3.8. Kyoto Encyclopedia of Genes and Genomes (KEGG) Pathway Enrichment Analysis of DEGs

**Figure 5:**
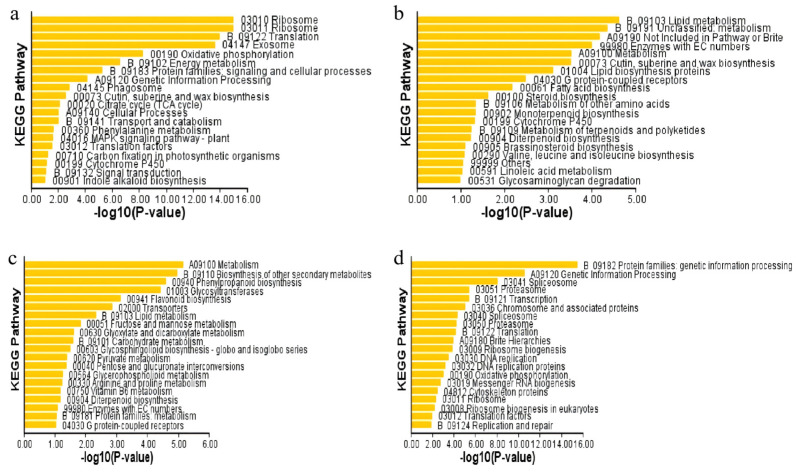
The top 20 KEGG terms of DEGs in *z047* and WT. (**a**) *z047* under drought stress for 7 days; (**b**) WT under drought stress for 7 days; (**c**) *z047* under drought stress 14 days; (**d**) WT under drought stress for 14 days.

Previous studies on the responses of plants to drought stress have shed new insights in understanding of the mechanisms of drought tolerance in different species. A number of protein families in the calcium signalling pathways, mitogen-activated protein kinases (MAPKs) signalling pathways and phosphorylation cascades were also involved in responses to drought stress [44,45]. In this study, the KEGG pathway enrichment analysis verified that under drought stress for 7 days, the DEGs were mainly enriched in ribosomes, translation, exosomes, and oxidative phosphorylation pathways in *z047* plants (Figure 5a), whereas those were chiefly enriched in lipid metabolism, metabolism, and EC-containing enzyme pathways in WT (Figure 5b). Under drought stress for 14 days, the DEGs were largely rich in metabolism, biosynthesis of other secondary metabolites, and phenylpropanoid biosynthesis pathways in *z047* (Figure 5c), while those were mostly enriched in genetic information processing, protein families: genetic information processing, translation, and ribosome pathways in WT plants (Figure 5d). All these data suggest that there were remarkable differences in the expression levels of DEGs in the different phases of drought-tolerant plants.

**Figure 6:**
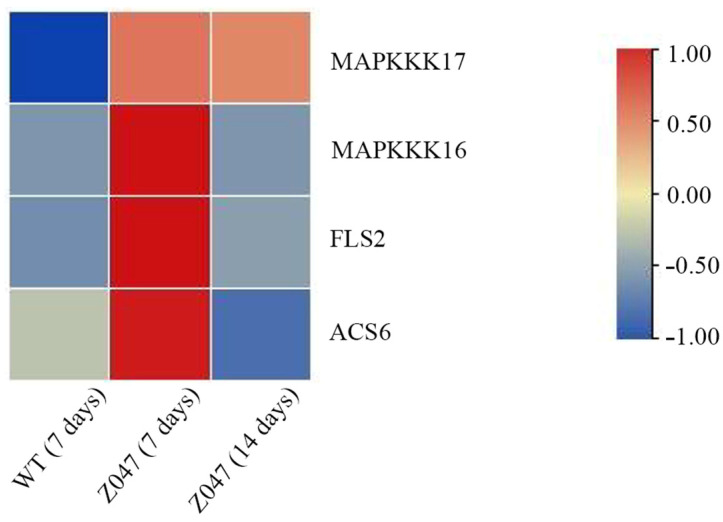
Gene analysis of MAPK metabolic pathway. WT (7 days), wild type plants under drought stress for 7 days; *z047* (7 days), *z047* plants under drought stress for 7 days; *z047*.

After exposure to drought stress for 7 or 14 days, gene analysis suggested that compared with WT plants, the highest transcription levels of *MAPKKK17*, *MAPKKK16*, *FLS2*, and *ACS6* genes in the mitogen-activated protein kinases (MAPKs) signalling pathways were in *z047* under drought stress for 14 days (Figure 6).

## 4. Conclusions

Based on the integrated physiological and transcriptome analysis, the study systematically analyzed the potential drought response mechanism underlying the mutant *z047*. The drought-resistant physiological experiments further confirmed that *z047* owns comprehensive drought tolerance, as demonstrated by a stronger water retention ability, lower membrane damage, and higher biomass accumulation under drought conditions, and its performance is superior to WT. Mechanismly, *z047* plants significantly improve their drought resistance by activating ribosome synthesis, oxidative phosphorylation, and phenylpropanoid metabolic pathways. Simultaneously, key genes, such as *MAPKKK17* and *MAPKKK16*, in the MAPK signalling pathway may enhance drought resistance by regulating cellular stress response. Collectively, this study reveals for the first time the multidimensional regulation of drought resistance in Japanese knotweed, providing important targets for subsequent functional gene validation and molecular marker-assisted breeding. At the same time, it provides scientific support for lawn establishment and ecological restoration practices in arid areas. Nevertheless, future research should further explore the regulatory mechanisms of key genes and their practical application potential in field environments.

## Data Availability

The original contributions presented in this study are included in the article. Further inquiries can be directed to the corresponding author.
